# Analysis of the Stability of Compounded Vancomycin Hydrochloride Oral Solution: A Comparison Between Formulations Prepared Using a Commercial Product and Using the Pure Active Ingredient

**DOI:** 10.7759/cureus.91993

**Published:** 2025-09-10

**Authors:** Jose David Arroyo-Solorzano, Stefanny Martínez-Carpio, Jeimy Blanco-Barrantes, Jorge Pacheco-Molina, Arlene Loría-Gutiérrez, Sebastián Arguedas-Chacón, Esteban Zavaleta-Monestel

**Affiliations:** 1 Pharmacy Department, Hospital Clínica Bíblica, San José, CRI; 2 Laboratory of Pharmaceutical Analysis and Consulting (LAYAFA), Faculty of Pharmacy, University of Costa Rica, San José, CRI; 3 Institute of Pharmaceutical Research (INIFAR), Faculty of Pharmacy, University of Costa Rica, San José, CRI; 4 Research Department, Hospital Clinica Biblica, San José, CRI

**Keywords:** drug compounding, drug stability, formulation design, high-performance liquid chromatography (hplc), physicochemical properties

## Abstract

Background

Oral vancomycin is the mainstay treatment for *Clostridioides difficile* infections. Compounded oral solutions are commonly prescribed, but their beyond-use date (BUD) is restricted to 14 days. Extending this limit would enhance patient convenience and pharmacy efficiency. Stability may depend on storage conditions and whether the formulation is prepared from pure active pharmaceutical ingredient (API) or a commercial injectable product.

Aim

To evaluate and compare the physicochemical and microbiological stability of compounded vancomycin hydrochloride oral solutions prepared from pure API and a commercial injectable product.

Methods

Two 12.5 mg/mL vancomycin hydrochloride formulations in United States Pharmacopeia (USP) Simple Syrup were prepared: one from Vitalis® injectable powder and one from pure API buffered with citric acid. Solutions were stored in high-density polyethylene (HDPE) bottles at room temperature (20°C) or refrigerated (2-8°C) for 90 days. Stability was assessed by high-performance liquid chromatography (HPLC), pH monitoring, and microbial testing according to USP <61> (microbial enumeration) and USP <62> (tests for specified microorganisms).

Results

Both formulations remained microbiologically stable for 90 days, with microbial counts within USP limits and no *Escherichia coli* detected. At room temperature, the API formulation lost >10% potency after 60 days, while the commercial product remained above 90% potency but showed >10% degradation from its initial value, with precipitation occurring by day 90. Under refrigeration, both formulations maintained potency and physical stability throughout the 90-day period. Buffering with citric acid preserved pH within USP specifications, whereas the unbuffered commercial product exceeded acceptable limits over time.

Conclusion

Compounded vancomycin hydrochloride oral solutions are stable and microbiologically safe for up to 90 days under refrigeration, and for at least 30 days at room temperature. Buffering agents are recommended to prevent pH drift and precipitation. Extending the BUD supports more flexible dispensing, reduces patient burden, and optimizes pharmacy workflows while ensuring therapeutic efficacy and safety.

## Introduction

Over the past decade, the incidence of *Clostridioides difficile* infection (CDI) has increased significantly, making it one of the most common infections acquired or treated both in hospital and community settings. Consequently, CDI has become a pressing public health concern [1]. *C. difficile* colonizes and proliferates in the human intestinal tract when the normal microbiota is disrupted, typically due to antibiotic use, leading to infectious diarrhea [2]. In the absence of effective treatment, CDI may progress to pseudomembranous colitis. Currently, the antibiotics of choice are oral vancomycin and fidaxomicin, with oral vancomycin being the most cost-effective option for healthcare systems [3]. 

Vancomycin is a glycopeptide antibiotic that inhibits the synthesis of bacterial cell walls in Gram-positive organisms, including *C. difficile*. Although poorly absorbed orally, it demonstrates effective local activity in the gastrointestinal tract [4]. Its mechanism of action involves acting as a structural analog of glycopeptide synthetase, binding via hydrogen bonds to the terminal D-alanine residues of the bacterial pentapeptide. This interaction disrupts transglycosylation and transpeptidation processes essential for peptidoglycan assembly [4]. Moreover, vancomycin molecules form homodimers, increasing structural rigidity and creating steric hindrance that impairs elongation of the peptidoglycan chain [5]. 

Although vancomycin has been in clinical use since 1956, its use is now recommended only under specific indications - particularly when resistance to other antibiotics is present or when patients have allergies to beta-lactams. In hospital settings, due to the frequent recurrence of CDI, oral vancomycin is often dispensed as a compounded preparation [3].

A study conducted by the University of Nottingham demonstrated that CDI managed at the inpatient level responds favorably to treatment with oral vancomycin and metronidazole following appropriate diagnosis [6]. According to the Society for Healthcare Epidemiology of America (SHEA) and the Infectious Diseases Society of America (IDSA), vancomycin is considered the first-line treatment, with recommended dosages of 125 mg, 250 mg, or 500 mg every six hours, either as monotherapy or in combination with various metronidazole regimens [7]. 

The therapeutic advantage of compounded preparations lies in their customized formulation, tailored to medical prescriptions that often require dosage adjustments based on individual patient needs. This represents a professional commitment to resolving access or health-related issues related to pharmaceutical form [8]. Compounded medications often involve modifications, such as changes in dosage form, removal or substitution of excipients and preservatives, and organoleptic improvements (e.g., color, flavor, odor) to enhance patient acceptance, particularly in vulnerable populations like children and the elderly [9].

For compounded pharmaceutical preparations, shelf life is generally determined based on beyond-use dates (BUDs) as outlined in pharmacopoeial monographs, official compendia such as United States Pharmacopeia (USP) <795>, or existing stability studies [9]. To establish an accurate BUD, factors such as dosage form, administration route, water content, storage conditions (temperature, humidity, light exposure), and the integrity of primary packaging must be considered [10]. These factors directly influence a product’s ability to maintain chemical, physical, and microbiological stability within acceptable quality parameters, ensuring both safety and therapeutic efficacy [10]. 

According to the reviewed scientific literature, compounded oral vancomycin solutions exhibit a refrigerated stability of up to 14 days [11]. In our hospital setting, data supporting extended stability would be highly beneficial, as compounded preparations are not only administered immediately but also dispensed to ambulatory patients whose treatment regimens often exceed 14 days. Many of these patients cannot return to the hospital daily, making it essential to dispense the full course of therapy at once. Furthermore, assessing the effect of storage conditions may justify more practical transportation methods, including those that do not require refrigeration. 

This study aims to evaluate the stability of a compounded vancomycin formulation over a 90-day period under both refrigerated and room temperature conditions. The goal is to assess the potential for extending the preparation’s shelf life beyond 14 days. Additionally, we analyzed active pharmaceutical ingredient (API) content and microbiological changes in two formulations: one prepared from an injectable form and another using pure vancomycin hydrochloride API with a more acidic pH. All chemical and microbiological analyses were conducted at the Laboratory of Pharmaceutical Analysis and Consultancy (LAYAFA) at the Faculty of Pharmacy, University of Costa Rica, which provides the necessary equipment and reagents for robust testing.

## Materials and methods

Sample preparation**

Two oral vancomycin hydrochloride formulations were prepared at the hospital’s compounding pharmacy. One formulation used a commercial lyophilized powder for injection (Vitalis®, Bogotá, Colombia), while the other used the pure active pharmaceutical ingredient (API) in powder form (Huichem, Shanghai, China). Both preparations were compounded to achieve a final concentration of 12.5 mg/mL of vancomycin hydrochloride using water for injection and Simple Syrup USP (Medisca Pharmaceutique Inc., St. Laurent, Quebec, Canada) as the vehicle. This concentration was selected to allow the preparation of 10 mL bottles containing 125 mg of vancomycin, enabling single-dose administration in line with the most common oral dosing regimen. For each formulation, a total of 400 mL of solution was prepared. 

To prepare the solution from the commercial injectable form, 10 vials of lyophilized vancomycin were each reconstituted with 10 mL of sterile water for injection. The reconstituted solutions were transferred to a glass beaker, combined, and mixed with 100 mL of Simple Syrup USP. The final volume was adjusted using sterile water for injection in a pharmaceutical-grade conical graduated cylinder and mixed thoroughly to ensure complete dissolution. 

The second formulation, prepared from the pure API, involved weighing 5.126 grams of vancomycin hydrochloride powder and combining it with 0.800 g of anhydrous citric acid (Medisca Pharmaceutique Inc.) and 100 mL of Simple Syrup USP in a glass beaker. As with the first formulation, the volume was brought to 400 mL with sterile water for injection and stirred until homogeneous. 

The Simple Syrup USP used in both formulations contained 850 g of sucrose per liter, sodium benzoate as a preservative, and citric acid as an acidifier. 

Storage and distribution 

Each solution was aliquoted into high-density polyethylene (HDPE) plastic bottles with screw caps. For each formulation, 32 bottles were prepared: eight bottles of 20 mL for microbiological testing and 24 bottles of 10 mL for physicochemical analysis. The volume difference was based on the testing requirements; however, the content in each bottle was identical. From each formulation, four microbiological and 12 physicochemical bottles were stored under refrigeration (2-8°C), while the remaining bottles were stored at controlled room temperature (20°C) to allow for comparative analysis.

Analytical procedures

For each microbiological test, 60 mL of solution was required, while 10 mL was used for physicochemical evaluation. At each testing time point, 1 mL aliquots were collected in triplicate from both formulations and analyzed using high-performance liquid chromatography (HPLC). Prior to analysis, samples were diluted with 25 mL of purified water and filtered through 0.45 μm membrane filters.

The following parameters were evaluated: 

(a) Organoleptic properties: appearance, color, presence of precipitation, and detection of foreign particles.

(b) pH measurement: assessed using a calibrated pH meter (pH 2700, Oakton, Singapore).

(c) These evaluations were conducted at the following time points: day 0 (baseline), and after 4, 14, 30, 45, 60, 75, and 90 days. 

Quantitative analysis of vancomycin 

*Chromatographic System* 

Quantification of vancomycin was performed using gradient HPLC on an LC 300 system (PerkinElmer, Waltham, MA, USA). The mobile phase consisted of two solutions: Solution A-50 mM ammonium acetate adjusted to pH 8.0 with ammonia, and Solution B, a methanol-purified water mixture (35:65). The detector was a UV/Vis deuterium lamp set at 280 nm. Chromatographic separation was achieved using an L1 column (2.1 mm × 25 cm, 5 µm particle size) at 40°C, with a flow rate of 1 mL/min and an injection volume of 10 µL [11]. Certified impurity standards for vancomycin were not commercially available in our study region; therefore, forced degradation studies (acidic, basic, oxidative, and photolytic conditions) were performed to evaluate the specificity of the method. Although certain degradation pathways and crystalline degradation products of vancomycin have been described in the literature, no certified reference materials were accessible. Consequently, literature-reported degradation profiles were used as supporting references during method validation.

Validation 

**Figure 1:**
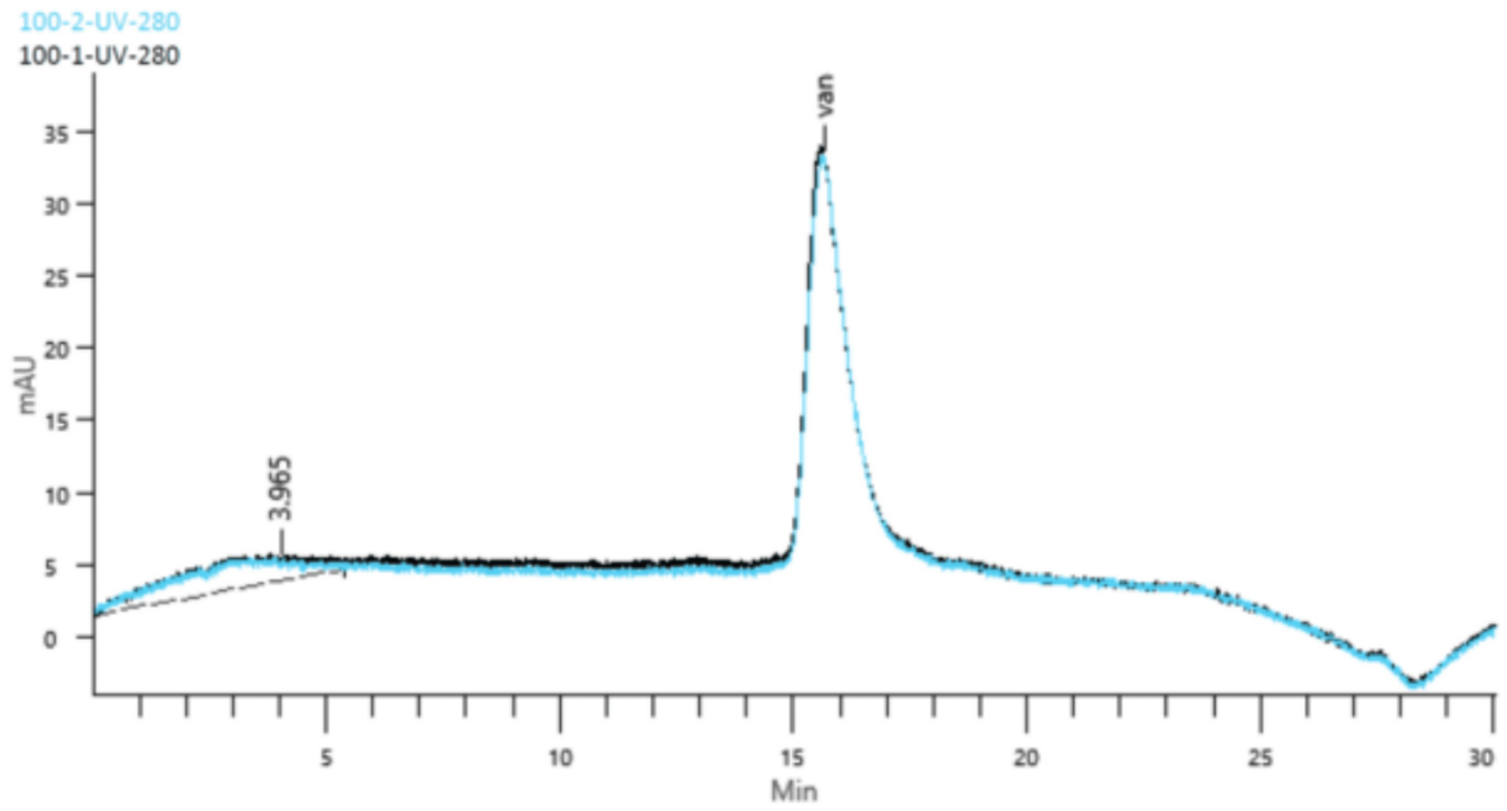
Chromatogram of the reference standard superimposed on the chromatogram of a test formulation sample

Due to the unavailability of certified impurity and degradation product standards for vancomycin, specificity was evaluated through forced degradation studies. Reference substance samples were subjected to ultraviolet light (5 h), heat (65°C for 48 h), acid hydrolysis (2 M HCl), alkaline hydrolysis (1 M NaOH), and oxidation (10 vol H₂O₂), and chromatograms were compared with those of samples prepared under normal conditions. The matrix of the oral preparation (placebo) was also analyzed. Heat-treated samples showed two additional peaks corresponding to vancomycin-related compounds 1 and 2, with resolution values of 3.1 and 4.2, respectively, which did not interfere with the vancomycin peak. No interfering peaks were observed in UV, oxidative, acidic, basic, or placebo samples, confirming the specificity of the method (Figure 1).

**Figure 2:**
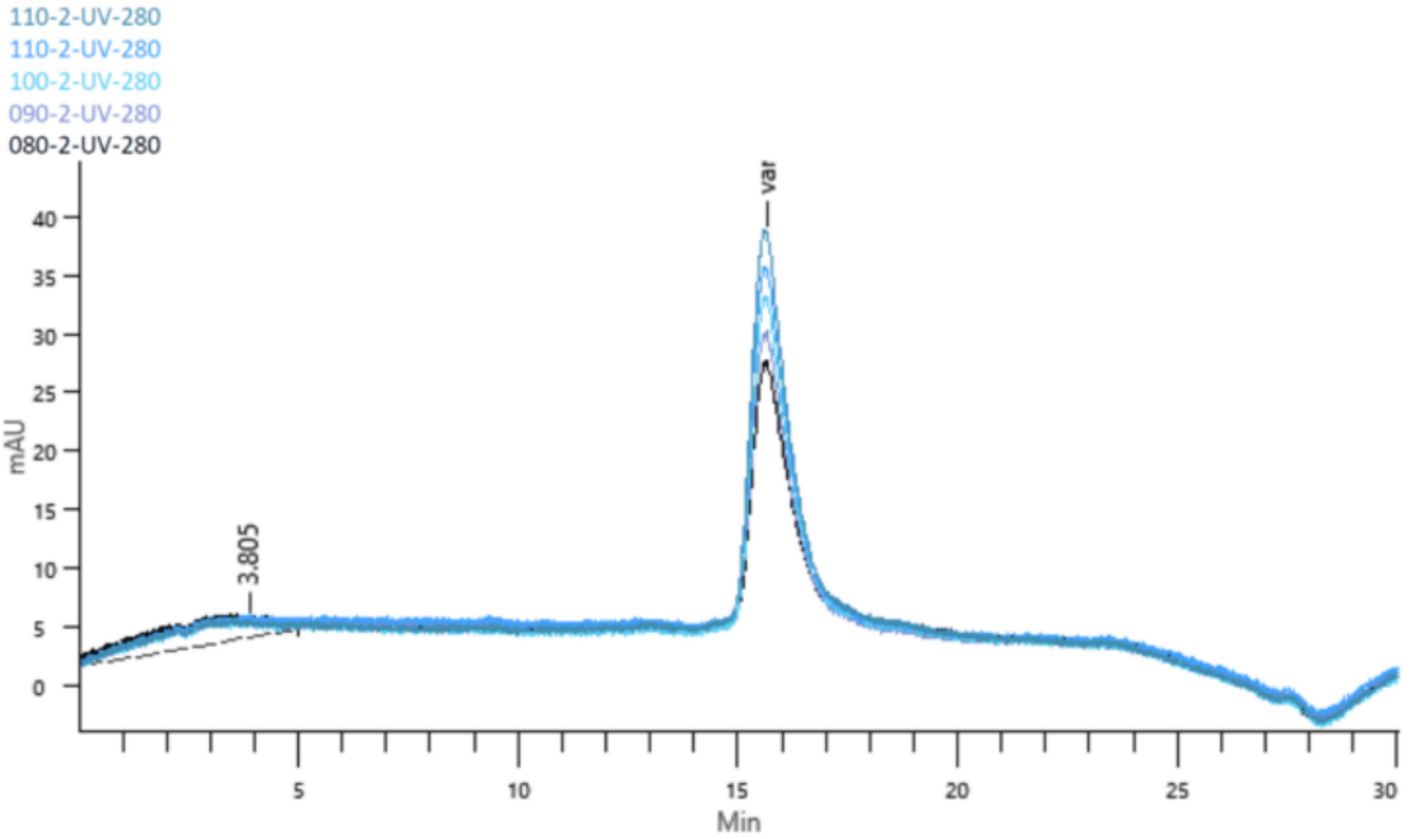
Chromatograms from the linearity test

Linearity was confirmed using five concentrations (80%-120% of test concentration) prepared from three independent stock solutions. A correlation coefficient (R²) of 0.996 was obtained. The 95% confidence interval for the y-intercept (−21.88 to 114.56) included zero, and the experimental t-value (1.47) was below the critical t-value (2.16), indicating no significant deviation. Residual analysis showed randomness with no systematic trend (Figure 2).

Precision was evaluated as repeatability using nine determinations (three concentrations with three replicates each) across the validated range. The relative standard deviation (RSD) was 1.1%, below the 2.0% acceptance criterion.

Accuracy was assessed using the same nine determinations. The mean recovery was 100.1% (within the 98.0-102.0% range), with a relative error of 0.7%, meeting acceptance criteria.

Preparation of the standard solution 

A standard vancomycin hydrochloride solution was prepared at a concentration of 0.5406 mg/mL, calculated gravimetrically as vancomycin B. For standard preparation, 0.029 g of vancomycin HCl raw material (Huichem) was dissolved in 25 mL of purified water (triplicate), and 0.029 g of a primary standard (Sigma Aldrich, St. Louis, MI, USA) was similarly prepared (duplicate). The Sigma Aldrich standard served as a reference to validate the raw material standard.

All solutions were prefiltered using a 0.45 µm membrane and sonicated for 5 min. A 1 mL aliquot of each was used to calibrate the method via external standardization. System suitability was confirmed by injecting six standard solutions at the beginning and three at the end of the analysis session. The potency of vancomycin was calculated by comparing the area ratio of the sample and standard chromatograms. Acceptance criteria were based on the United States Pharmacopeia (USP): not less than 90.0% [11].

Microbial count test for a non-sterile product

Membrane Filtration and Plate Counting Method

Microbiological quality testing of the oral vancomycin formulations was conducted according to USP Chapters <61> (Microbial Enumeration Tests) and <62> (Tests for Specified Microorganisms). As specified in Chapter <61>, a growth promotion test was performed on all culture media, and a microbial enumeration proficiency test was conducted using the following reference strains: *Staphylococcus aureus *ATCC 6538,* Pseudomonas aeruginosa* ATCC 9027, *Bacillus subtilis* ATCC 6633, *Candida albicans* ATCC 10231, and* Aspergillus brasiliensis* ATCC 16404. In compliance with Chapter <62>, suitability for detecting *Escherichia coli* was confirmed using strain ATCC 8739.

Each test used 10.0 mL of solution from both formulations and storage conditions. All samples were analyzed in duplicate. Membrane filtration was carried out after washing the membrane with Flushing Fluid K (composed of 5.0 g peptic digest of animal tissue, 3.0 g beef extract, and 10.0 g polysorbate 80), adjusted to pH 7.2 with phosphoric acid, filtered, and sterilized via autoclaving (Tuttnauer 3850E). 

Each bottle was vortexed, and 10.0 mL was diluted in 90 mL of tryptic soy broth with lecithin and polysorbate 80. The entire volume was vacuum filtered through a 0.45 µm membrane filter, followed by five washes with 100 mL of Flushing Fluid K. This process neutralized the antimicrobial activity of vancomycin. 

For microbial count proficiency, a 1:10 dilution of the sample was prepared in casein-soybean digest broth with lecithin (0.7 g/L) and polysorbate 20 (5 g/L). A 10 mL aliquot (equivalent to 1 mL of sample) was filtered through 0.45 µm, 5 mm nitrocellulose membranes. After five rinses with Flushing Fluid K, the membranes were inoculated with <100 CFU of each strain. They were then placed on casein-soy digest agar (for bacteria) or Sabouraud dextrose agar (for fungi). Incubation was as follows: bacteria for 24 hours at 32.5±2.5°C (IC603CR, Yamato Scientific America, Santa Clara, CA, USA), *Candida* for 48 hours, and *Aspergillus* for 96 hours at 22.5±2.5°C (KT 170, Bider GmbH, Tuttlingen, Germany). Colony counts were compared with untreated controls. 

Specific Microorganism Detection

For *E. coli* detection, 10 mL of the sample was diluted in 90 mL of casein-soy digest broth with lecithin and polysorbate 20. A 100 mL portion (10 mL of sample equivalent) was filtered, washed five times with Flushing Fluid K, and inoculated with <100 CFU of *E. coli* ATCC 8739. Each membrane was incubated in 90 mL of broth at 32.5±2.5°C for 24 hours. Subsequently, 1.0 mL of this culture was transferred to MacConkey Broth and incubated for 24 hours at 42°C (Blue M 100A, ZA-2950), followed by subculture on MacConkey Agar for another 24 hours at 32.5°C (Blue M 200A, ZA-5907).

Acceptance Criteria 

According to USP guidelines, microbial limits for non-sterile oral preparations are as follows: no more than 200 CFU/mL for bacteria, no more than 20 CFU/mL for fungi and molds, and absence of *E. coli.* per mL of product [12].

## Results

HPLC analysis

The retention time of the main peak in the chromatograms of all test samples coincided with that of the vancomycin reference standard, confirming identity and method specificity. Quantitative analysis, based on the average of three measurements, showed that the standard solution contained 92.2% of the expected vancomycin content, with a standard deviation of 2.3% and an RSD of 2.5%, within a 95% confidence interval.

Organoleptic properties

The formulation prepared from the commercial injectable exhibited a clear and transparent appearance, with no precipitation or visible foreign particles, and had a faint yellow color. The formulation made from the pure API was similarly transparent and free of particulate matter, but had a slightly deeper yellow hue, attributable to the pinkish tone of the raw vancomycin powder, in contrast to the white lyophilized powder of the injectable.

These visual characteristics remained unchanged throughout the 90-day stability study for all samples, except the injectable-derived sample stored at room temperature, which presented visible precipitation at day 90, indicating loss of physical stability.

Microbiological quality

All tested samples met USP acceptance criteria for microbial counts and absence of *E. coli *[12]. Recovery rates for microbial enumeration were within acceptable ranges: 76%-133% for the pure API formulation and 65%-109% for the injectable formulation, consistent with USP acceptance criteria (50-200%). The *E. coli* strain was successfully recovered in positive controls but was absent in all vancomycin-containing samples, confirming microbial safety.

Formulation stability

The compounded formulation from the pure API included citric acid to regulate pH within USP specifications. The buffering effect of the citrate ion stabilized the solution’s acidity, ensuring appropriate formulation conditions [13].

As presented in Table 1, initial API content was 107.6% for the injectable formulation and 95.6% for the formulation made from the pure API. The higher content in the injectable-derived solution may be due to the lyophilized nature of the powder, which offers enhanced chemical stability. Moreover, lyophilized vials provide vacuum-sealed packaging, preserving drug potency better than powdered APIs stored in bulk. It should be noted that, unlike in the pharmaceutical industry, quality control testing of raw material upon receipt is not feasible in a hospital compounding setting. Additionally, the gravimetric factor used did not include a correction for assay value or moisture content.

**Table 1:** Percentage of vancomycin content recovered over time under different storage conditions Room Temp.: 20°C; Refrigerated: 2-8°C; Commercial drug; API=active pharmaceutical ingredient; NA=not available (no measurement performed or sample not analyzable).

Day	Commercial Drug (CD)		Pure Active Ingredient (API)	
	Room Temp. (%)	Refrigerated (%)	Room Temp. (%)	Refrigerated (%)
0	107.6±1.3	NA	95.6±1.3	NA
14	104.7±0.7	109.5±1.4	92.8±0.4	97.1±1.4
30	104.8±1.6	109.7±0.4	90.9±0.4	97.8±0.8
60	97.9±2.3	109.5±0.0	84.3±2.0	98.3±0.8
90	NA	109.6±0.9	73.6±0.4	95.2±0.3

On day 14, samples stored under refrigeration retained higher vancomycin content than those stored at room temperature. Specifically, the injectable-derived formulation contained 109.5% under refrigeration versus 104.7% at room temperature. The pure API formulation retained 97.1% refrigerated and 92.8% at room temperature. All values remained within USP specifications (not less than 90.0% and not more than 110.0%) [11]. According to compounding stability criteria, an additional requirement is that content should not vary by more than ±10% from the initial value; this was applied when interpreting results.

By day 30, a decline in vancomycin content was observed, particularly in samples stored at room temperature. The pure API formulation approached the lower specification limit. At day 60, the room-temperature-stored pure API formulation dropped to 84.3%, falling below acceptable limits and indicating significant degradation. Refrigerated samples of both formulations, however, remained within specifications.

The injectable-derived formulation stored at room temperature retained 97.9% content at day 60, essentially the same as at day 30 (104.8%), but considering its initial value of 107.6%±1.3%, a degradation greater than 10% was evident. Therefore, although technically within labeled content specifications, degradation relative to time zero exceeded acceptable thresholds for compounded stability, rendering the solution unsuitable for patient use at day 60.

At day 90, HPLC analysis could not be performed for the injectable-derived sample stored at room temperature due to precipitation, indicating a loss of physical and chemical stability. The remaining samples were analyzable, and API content continued to decline in those stored at room temperature.

To determine whether the degradation observed in the pure API formulation was statistically significant, a two-tailed Student’s t-test for independent samples (n=3 per time point) was performed. The experimental t-value (2.8611) exceeded the critical value (2.1318) at a 95% confidence level, confirming a significant reduction in API content over time under room temperature storage conditions.

Microbiological stability

The proficiency test for both microbial enumeration and specific microorganism detection confirmed the validity of the analytical method. Recovery of test strains was successful, and antimicrobial neutralization during sample handling was effective. Throughout the 90-day period, no sample showed contamination with *E. coli*, fulfilling USP requirements for non-sterile oral preparations [12].

Aerobic microbial counts remained at 0 CFU/mL in all samples until day 60. At that point, all samples showed minimal growth, ranging from 1 to 4 CFU/mL. Gram staining identified the presence of Gram-positive bacilli. These low counts are within acceptable safety margins, and the formulations were still considered suitable for human use.

At day 90, only the room-temperature-stored pure API formulation showed microbial growth (3 CFU/mL), while all other samples remained sterile.

Table 2 summarizes the presence of fungi and yeasts detected in compounded vancomycin hydrochloride oral solutions, prepared either from the CD or from the pure API, and stored under room temperature or refrigeration for up to 90 days.

**Table 2:** Colony-forming units (CFU)/mL found by microbial count test for aerobic bacteria and Escherichia coli in vancomycin HCl samples from the commercial drug product and the pure active substance Room Temp.: 20°C.; Refrigerated: 2-8°C; CD=Commercial drug; API=active pharmaceutical ingredient; CFU=colony-forming units; NA=Not available (no measurement performed or sample not analyzable)

Day	Aerobic Bacteria (CFU/mL)				*Escherichia coli* (CFU/mL)			
	Room Temp. – CD	Room Temp. – API	Refrigerated – CD	Refrigerated – API	Room Temp. – CD	Room Temp. – API	Refrigerated – CD	Refrigerated – API
0	0	0	NA	NA	0	0	NA	NA
30	0	0	0	0	0	0	0	0
60	4	2	1	3	0	0	0	0
90	0	1	0	0	NA	0	0	0

Fungal contamination

As shown in Table 3, fungal growth was observed on day 30 in the refrigerated samples of both formulations. The injectable-derived formulation presented 5 CFU/mL of *Penicillium* species, while the pure API formulation showed 1 CFU/mL of yeast. These findings were isolated and not replicated in other samples, suggesting possible environmental contamination during sample handling. Bottles used were not sterile and were exposed to environmental particles during filtration or due to their closure mechanism (cap seal without full cover), increasing contamination risk.

**Table 3:** Fungal and yeast counts (CFU/mL) in vancomycin HCl oral solution samples Room Temp.: 20°C.; Refrigerated: 2-8°C; CD=commercial drug; API=active pharmaceutical ingredient; CFU=colony-forming units; NA=not available (no measurement performed or sample not analyzable)

Day	Fungi and Yeast (CFU/mL)			
	Room Temp. – CD	Room Temp. – API	Refrigerated – CD	Refrigerated – API
0	0	0	NA	NA
30	0	0	5 (*Penicillium* sp.)	1 (*Yeast*)
60	1 (*Yeast*)	0	0	0
90	NA	0	NA	0

Despite this, fungal contamination remained within USP limits for yeasts and molds, and the solutions were deemed acceptable for consumption. At day 60, one sample of the room-temperature-stored injectable formulation showed 1 CFU/mL of yeast. No fungal growth was observed in any sample by day 90.

Influence of pH

Vancomycin is classified as a weak base, with several ionizable functional groups including β-hydroxychlorotyrosine units, substituted phenylglycine rings, amide bonds, and a disaccharide composed of glucose and vancosamine, its characteristic amino sugar [4]. These chemical characteristics confer greater molecular stability in acidic environments. While the ideal pH range for vancomycin in solution is 2.0 to 4.0, the USP specifies a narrower acceptable range between 2.5 and 3.5.

In formulations lacking pH adjustment, such as the one prepared from the commercial injectable product, a gradual pH increase was observed over time. At day 46, the pH of this solution reached 3.6, exceeding the USP limit. By day 90, this increase corresponded with visible precipitation, signaling a loss of physical stability. This behavior suggests that pH drift over time can be detrimental to the formulation’s structural integrity.

**Figure 3:**
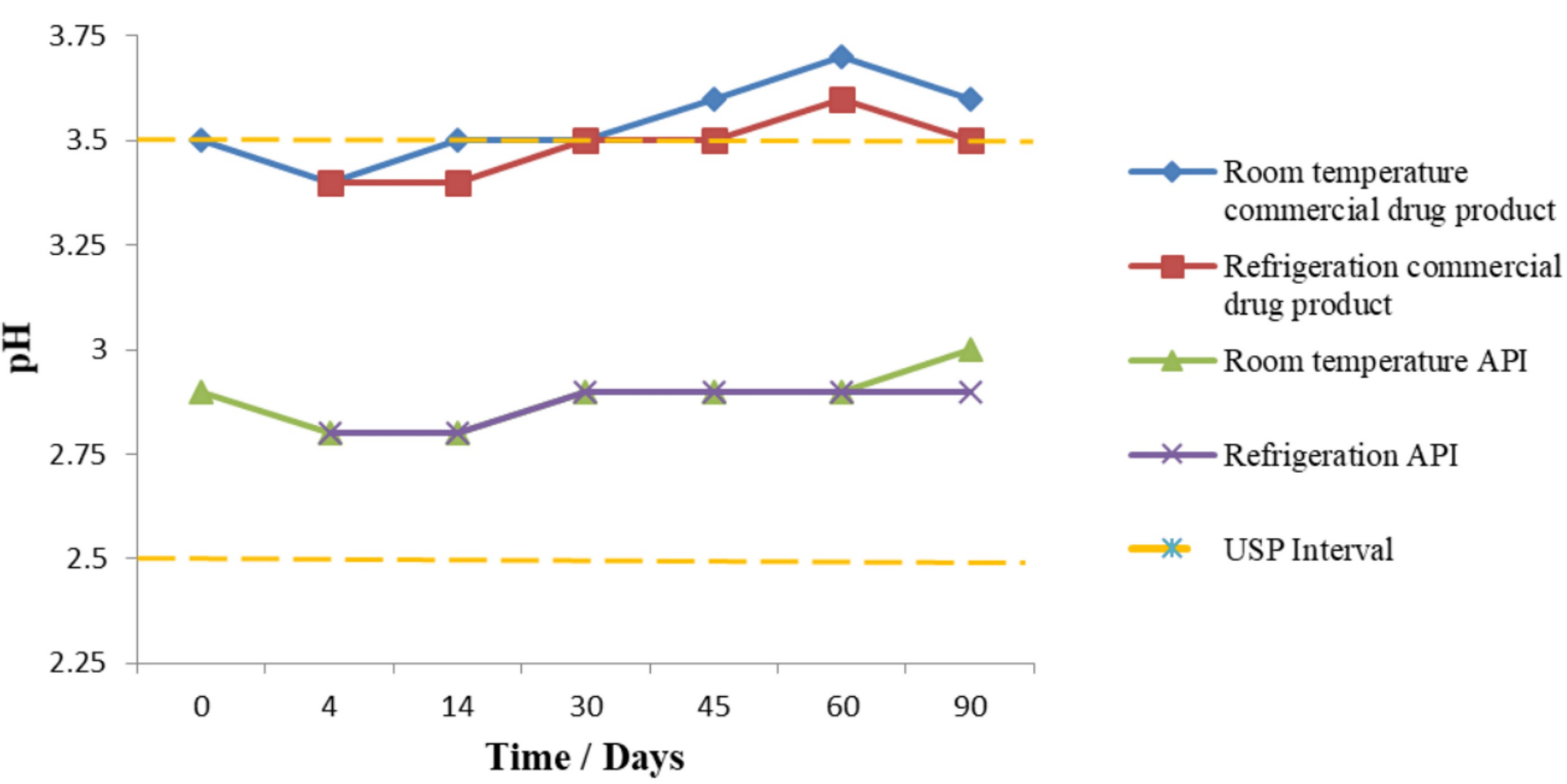
Effect of storage time and temperature on pH stability of compounded vancomycin formulations

In contrast, the formulation prepared from the pure vancomycin hydrochloride API included citric acid as a buffering agent. As shown in Figure 3, the addition of citric acid successfully maintained the pH within the USP-specified range under both storage conditions (refrigeration and room temperature). Even though a slight increase in pH was observed starting at day 60 in both formulations, the API-based formulation remained within specification throughout the 90-day period.

These results indicate that while pH changes may not directly affect the quantitative stability of the active ingredient (as API content remained within specification during the early phase of pH elevation), they are critical for ensuring physical stability. Precipitation due to pH rise may compromise drug uniformity and safety, reinforcing the importance of buffering agents in compounded formulations.

Influence of temperature

Storage temperature is a key determinant in the chemical stability of pharmaceutical preparations. As shown in Table 1, both formulations retained higher vancomycin content when stored under refrigeration (2-8°C), compared to samples stored at room temperature (20°C). This trend aligns with established literature recommending refrigerated storage for oral vancomycin solutions, although temporary exposure to 25°C is considered acceptable [14].

Previous work has also highlighted the relevance of pH and temperature as critical stability parameters in compounded oral liquid formulations [15]. The degradation of vancomycin is temperature-dependent, primarily due to hydrolytic processes that proceed more rapidly at higher temperatures [16]. Lower storage temperatures slow down the kinetics of such degradation reactions, preserving the integrity of the active compound for longer periods.

At day 60, both refrigerated formulations - regardless of the source of vancomycin - remained within USP specifications (content not less than 90.0% of label claim). In contrast, the formulation prepared from raw vancomycin hydrochloride and stored at room temperature fell below this threshold, indicating instability. By day 90, only the refrigerated samples continued to meet both physical and chemical stability criteria.

To confirm the significance of these findings, a two-tailed t-test was performed to compare API content between storage conditions for the API-based formulation. The mean potency at room temperature was 85.40%, whereas the refrigerated samples averaged 97.10%. The experimental t-value (2.6667) exceeded the critical value (2.4469), confirming a statistically significant difference at a 95% confidence level. These results clearly demonstrate that degradation occurs more rapidly at room temperature and validate the necessity of refrigerated storage for maintaining formulation stability.

## Discussion

The results of this study confirm that both storage temperature and formulation composition significantly influence the chemical, physical, and microbiological stability of compounded vancomycin oral solutions. The analytical method showed specificity, and the initial API content across all formulations complied with USP standards [11].

The inclusion of citric acid as a buffering agent in the API-based formulation proved essential to maintain pH within the USP-recommended range (2.5-3.5) [4], thereby preventing pH drift and precipitation. This observation is consistent with reports that identify pH control as a critical quality parameter in oral liquid compounding [15]. In contrast, the unbuffered injectable-derived formulation showed pH elevation over time, leading to precipitation and compromised physical stability.

Microbiological testing confirmed the formulations' safety throughout the study, despite minimal microbial growth at later time points. The isolated fungal contamination events on day 30 were most likely due to environmental exposure during filtration or bottle handling. This highlights the need for stricter aseptic techniques and improved handling practices in the compounding laboratory to further minimize the risk of contamination, rather than indicating intrinsic formulation failure [17]. All microbial values remained within USP acceptance limits [12].

Chemical stability was directly impacted by storage conditions. Refrigeration consistently preserved API content above the 90% threshold, whereas room-temperature storage led to accelerated degradation. A statistically significant difference in API content between storage conditions further validated these findings (p<0.05) [14,18]. These results align with other studies assessing vancomycin degradation rates in aqueous media, in which hydrolysis was more pronounced at higher temperatures and alkaline pH values [16].

Importantly, while the injectable-derived formulation retained API levels above 90% on day 60, the relative degradation exceeded 10% from the initial value (107.6%), which surpasses the typically accepted degradation threshold for compounded medications [13]. In this regard, several studies support limiting compounded vancomycin solutions to 14 or 30 days at room temperature in the absence of validated extended stability data [19]. Furthermore, extemporaneous formulations of vancomycin in alternative dosage forms - such as ophthalmic solutions or infusion syringes - have also demonstrated pH sensitivity and degradation patterns influenced by temperature and excipient composition [20,21]. Lastly, even vancomycin solutions for continuous infusion must adhere to strict compatibility and stability criteria, underscoring the broader relevance of these findings beyond oral administration [22].

## Conclusions

This study demonstrated that the BUD of compounded vancomycin hydrochloride oral solutions can be safely extended from 14 to 90 days when stored under refrigeration. Both formulations - prepared from pure API and the commercial injectable product - maintained chemical and microbiological stability for up to 90 days at 2-8°C. At room temperature, both formulations remained stable for up to 30 days; however, the commercial product exhibited precipitation by day 90, indicating a loss of physical stability.

Microbiological testing confirmed the absence of *E. coli *and acceptable microbial counts throughout the study period. Detected microbial growth was minimal and attributed to possible external contamination during handling or filtration. These findings underscore the importance of aseptic techniques during preparation and the use of clean, sterile primary packaging components.

It is therefore recommended that compounded vancomycin oral solutions be stored under refrigeration and prepared within a biosafety cabinet when possible. Proper sanitation of primary containers should also be ensured to maintain product integrity and patient safety.
